# Imaging dissociation in post-traumatic stress disorder

**DOI:** 10.1192/bjo.2023.25

**Published:** 2023-02-22

**Authors:** Yann Quidé

**Affiliations:** NeuroRecovery Research Hub, School of Psychology, University of New South Wales, Sydney, New South Wales, Australia; and Centre for Pain IMPACT, Neuroscience Research Australia, Randwick, New South Wales, Australia

**Keywords:** Trauma, post-traumatic stress disorder, imaging, dissociative disorders, childhood experience

## Abstract

Symptom provocation paradigms have been successfully developed to identify the neural correlates associated with post-traumatic stress disorder (PTSD) symptoms, especially dissociative behaviours, but have critical limitations. Transiently stimulating the sympathetic nervous system and/or the hypothalamic–pituitary–adrenal (HPA) axis can enhance the stress response to symptom provocation and would help identify targets for personalised interventions.

The study by Mertens and colleagues published in the *BJPsych Open*^1^ is the largest (*n* = 51) study using functional magnetic resonance imaging (fMRI) while employing so-called script-driven imagery (SDI) in a relatively homogeneous group of female victims of childhood trauma. SDI is a symptom-provocation paradigm specifically designed to trigger post-traumatic stress disorder (PTSD) symptoms and adapted for fMRI experiments.^2,3^ Prior to the scanning session, participants are asked to recall both a neutral life event and their traumatic event. Neutral and traumatic scripts are derived from this narrative and integrated into the fMRI paradigm. In the scanner, participants are asked to passively listen to the scripts (retrieval) before actively recalling these autobiographical memories (re-experiencing). Severity of re-experiencing, avoidance and dissociative behaviours experienced during the task is measured after the scanning session, using the Responses to Script-Driven Imagery Scale (RSDI).^4^ Only a few brain imaging studies have investigated associations between brain function and dissociation in PTSD, reporting heterogeneous results.^5^

## Trauma-related dissociation

During trauma exposure, survivors often report dissociative experiences such as depersonalisation or derealisation.^6^ Dissociation is often used as a coping mechanism when exposed to traumatic events,^7^ and it contributes to the development of more severe forms of PTSD.^8^ Despite important advancement in theoretical modelling of post-traumatic dissociative behaviours, the identification of brain phenotypes associated with dissociation, especially in people with PTSD, remains challenging. In the study by Mertens and colleagues,^1^ the levels of dissociation recorded by the participants on the RSDI and the patterns of brain activation in response to SDI were similar to those reported in a previous independent study,^9^ including trauma-related increased activation in brain regions involved in autobiographical memory (amygdala, cerebellum, supramarginal and inferior occipital gyri). However, the authors reported no significant association between changes in brain function in response to the traumatic script (overall, or during any phase of the task) and reported dissociative symptoms. This was at odds with previously reported correlation between increased dissociation score and increased medial prefrontal activation.^9^ Follow-up region-of-interest analyses in the amygdala confirmed the patterns of activation in response to the script, as well as the lack of association with the dissociation scores for either the retrieval or re-experiencing phases.

## Study limitations and strengths

Several limitations, noted by the authors, should be highlighted as they may largely account for this lack of association. The first limitation is inherent in the paradigm used, which only allows detection of correlational associations between brain responses to SDI and self-reported (subjective) symptom severity recorded after the scanning session, prohibiting any causal inference. A second limitation is relative to the nature of the studied sample. Although it represents a relatively homogeneous group of females suffering from PTSD related to childhood trauma, which could arguably represent a strength, this population is different in terms of type, severity and chronicity of trauma exposure from samples in other studies (e.g. victims of motor vehicle accidents), making comparisons between studies difficult. In addition to the correlational nature of the study, the level of acute dissociation reported in response to the traumatic script was relatively low (average dissociation score of 2.4, ranging from 0 to 6, out of a maximum of 24 points for this domain). Although consistent with a previous study,^9^ the range of scores reported, relative to the overall range of the questionnaire, may have limited the amount of variation necessary to detect any meaningful association with changes in brain function: the severity of the symptoms reported may be overall too weak to induce strong and consistent changes in brain function. Finally, the authors noted that participants might have already been exposed to their trauma via participation in prior trauma-focused interventions that may have attenuated their responses to SDI.

These limitations should not undermine the importance of the study by Mertens and colleagues: it is one of the largest and most powered studies of its kind. As previously mentioned, a strength of this study is the relatively heterogeneous sample of females with PTSD related to childhood trauma exposure. Despite the lack of brain–behaviour association reported, this study indirectly suggests that other factors and/or mechanisms are likely to be at play when studying the functional brain changes associated with trauma-related dissociative behaviours. The authors noted that using self-reported severity of dissociative symptoms in conjunction with brain imaging techniques of high spatial (fMRI) and temporal resolutions (electroencephalogram, EEG) combined would be an important next step. Although this approach is necessary and will complement the current understanding of the neural circuits at play during symptom provocation, it will not address the limitations associated with the paradigm itself. Alternative paradigms used to enhanced the provoked PTSD symptoms and objective measures of these symptoms in brain imaging settings are needed.

## New paradigms to trigger dissociative behaviours in research

What paradigm would be able to trigger strong and safe enough levels of dissociation in research settings? SDI is the gold standard paradigm used to trigger PTSD symptoms and should remain core to future experiments. Additional sensory stimulations (e.g. touch) could be used to enhance the response triggered by the traumatic script. Although this would be feasible in clinical settings or when using EEG or psychophysiological experimental approaches, it may be perceived as distressing and would be challenging in MRI environments. Transiently stimulating the sympathetic nervous system and/or the hypothalamic–pituitary–adrenal (HPA) axis, using SDI or other interventions that can enhance the stress response or increase glucocorticoid receptor sensitivity, can strengthen the stress response to the traumatic script. These two systems are central to the neurobiology of dissociative behaviours and increasing their activation would lead to larger release of adrenaline and glucocorticoids (cortisol) and/or increase glucocorticoid receptor sensitivity, thus increasing the heart rate and triggering stronger dissociative behaviours in response to the traumatic script. This physiological change is critical to prepare the body to enter the ‘fight or flight’ response to the traumatic experience, additionally increasing the likelihood of experiencing dissociative behaviours.^10^ This approach would potentially induce transient, short and strong enough dissociative experience to show significant associated changes in brain function.

Within this context, future studies will need to develop new, or adapt existing, objective ways to measure the severity of dissociative behaviours in response to presentation of the SDI paradigm. Mertens and colleagues used the Clinician-Administered Dissociative States Scale (CADSS)^11–13^ in their study, both before and after the scanning session, as a measure of the participant's dissociative state occurring between measurements, but did not use CADSS scores in their imaging analyses. This scale is powerful as it includes both an interview that will capture the participant's experience (subjective, similar to SDI) and an external observation of dissociative behaviours (objective), although the objective part of the CADSS still needs some validation.^12^ In the sample presented by Mertens and colleagues, the dissociative part of SDI and the CADSS show relatively strong correlation (*r* = 0.46). In addition, the CADSS has a larger score range than the RSDI, potentially making it a good candidate to use with functional imaging data. Post- minus pre-traumatic script differences in CADSS scores could be used as an overall index of dissociative state during the presentation of the SDI traumatic condition and used in associations with changes in brain response to the traumatic script. It is important to note that this approach would not address the issue relative to the correlational nature of the analyses, inherent in most fMRI experiments, but it might be a more powerful methodological approach for this kind of experiment. Future studies are needed to investigate the relationship between state dissociation in the scanner as measured by the CADSS and brain response to the traumatic script, and to determine its suitability and sensitivity compared with the RSDI.

## Conclusions

Identifying the neural correlates of PTSD symptoms, especially dissociative behaviours, remains challenging, and the current approaches may not be optimal. Despite the potential advantages of the above-proposed approaches in triggering (e.g. enhancing the stress response or increase glucocorticoid receptor sensitivity) and measuring dissociative behaviours (e.g. using the CADSS), the identification of brain correlates of dissociation in PTSD remains correlational in nature, with fMRI paradigms not designed to identify causal associations. Real-time EEG recording would be an important addition to fMRI that might be able to identify the changes in brain function associated with the dissociative state. Together, these approaches will provide new avenues to our understanding of the neurobiological changes associated with PTSD symptoms, especially dissociation, and might identify targets for personalised interventions.

## Data Availability

Data availability is not applicable to this article as no new data were created or analysed in this study.
